# Disentangling the Relationship between Physician and Organizational Performance: A Signal Detection Approach

**DOI:** 10.1177/0272989X20936212

**Published:** 2020-07-01

**Authors:** Olga Kostopoulou, Martine Nurek, Brendan C. Delaney

**Affiliations:** Imperial College London, Department of Surgery and Cancer, London, UK; Imperial College London, Department of Surgery and Cancer, London, UK; Imperial College London, Department of Surgery and Cancer, London, UK

**Keywords:** colorectal cancer, conversion rate, detection rate, gender differences, primary care, QCancer, response bias, stress from uncertainty

## Abstract

**Background.** In previous research, we employed a signal detection approach to measure the performance of general practitioners (GPs) when deciding about urgent referral for suspected lung cancer. We also explored associations between provider and organizational performance. We found that GPs from practices with higher referral positive predictive value (PPV; chance of referrals identifying cancer) were more reluctant to refer than those from practices with lower PPV. Here, we test the generalizability of our findings to a different cancer. **Methods.** A total of 252 GPs responded to 48 vignettes describing patients with possible colorectal cancer. For each vignette, respondents decided whether urgent referral to a specialist was needed. They then completed the 8-item Stress from Uncertainty scale. We measured GPs’ discrimination (*d′*) and response bias (criterion; *c*) and their associations with organizational performance and GP demographics. We also measured correlations of *d′* and *c* between the 2 studies for the 165 GPs who participated in both. **Results.** As in the lung study, organizational PPV was associated with response bias: in practices with higher PPV, GPs had higher criterion (b = 0.05 [0.03 to 0.07]; *P* < 0.001), that is, they were less inclined to refer. As in the lung study, female GPs were more inclined to refer than males (b = −0.17 [−0.30 to −0.105]; *P* = 0.005). In a mediation model, stress from uncertainty did not explain the gender difference. Only response bias correlated between the 2 studies (*r* = 0.39, *P* < 0.001). **Conclusions.** This study confirms our previous findings regarding the relationship between provider and organizational performance and strengthens the finding of gender differences in referral decision making. It also provides evidence that response bias is a relatively stable feature of GP referral decision making.

Earlier detection, diagnosis, and treatment of cancer can save lives. Nevertheless, many barriers exist along the path from the physician suspecting cancer to treating it.^1^ The United Kingdom has long been lagging behind the best-performing countries in Europe in terms of cancer survival.^2^ This has been attributed in part to delays in diagnosis. Successive governments introduced measures to expedite the recognition and diagnosis of cancer symptoms. One of these measures is the 2-week-wait (2WW) referral pathway, or urgent referral pathway, whereby general practitioners (GPs) can refer a patient urgently to a specialist if they suspect cancer. The patient is then seen by the specialist within a target of 2 weeks. The 2WW referral pathway is fulfilling its purpose: a higher rate of urgent referrals is associated with lower mortality for common cancers and lower probability of late-stage diagnosis.^3^

The 2WW referral performance of UK general practices (family medicine clinics) is measured by 2 indices: the positive predictive value (PPV; i.e., the proportion of urgent referrals from a practice that result in a cancer diagnosis, also known as the “conversion rate”) and the sensitivity (i.e., the proportion of patients diagnosed with cancer who were urgently referred by their practice, also known as the “detection rate”). Although practices are not directly penalized or rewarded for their urgent referral performance, the data are monitored and available for audit and the commissioning of services. Furthermore, they are publicly available for scrutiny (https://fingertips.phe.org.uk/profile/general-practice).

There is substantial variation between practices in their referral performance, which previous research has attributed partly to the way the health care system is organized locally.^4^ The contribution of provider performance has not been possible to assess because of the lack of data about the referral performance of individual GPs. Despite this, an inference has been drawn from the organizational to the individual level by suggesting that high referral PPV and sensitivity are indicators of good clinical practice.^5^ Good clinical practice necessitates that GPs use information about risk factors and presenting symptoms appropriately to decide which patients are likely to suffer from cancer and that they use an appropriate threshold of cancer risk that minimizes the number of cancers missed without substantially increasing false-positive referrals. This cancer risk threshold is currently set at 3% by the National Institute for Health and Care Excellence (NICE; https://www.nice.org.uk/guidance/ng12/chapter/Introduction).

We have previously conceptualized the decision whether to refer a patient urgently for suspected cancer as a signal detection problem. GPs see several patients with symptoms that may suggest cancer but may also suggest other causes. GPs then have to decide whether there is sufficient evidence for an urgent referral, in other words, whether they think that the cancer “signal” is strong enough to justify a referral. GPs differ along 1 or both of the following dimensions: how they assess the presenting evidence (which is determined by both how uncertain the evidence is and their ability to assess it) and how much evidence they need before they refer. Signal detection theory (SDT)^6^ separates those 2 decision factors, known as “discrimination” and “response bias,” respectively, and proposes different methods for tackling each. SDT is based on statistical decision theory and thus requires respondents to make repeated decisions on multiple trials for their performance to be quantified. It follows that the experimenter needs to know the correct response on each trial. We should emphasize that the correct response in our study was not based on patient outcome (whether the patient has cancer or not) but on adherence to a national, risk-based guideline for urgent referrals for suspected cancer (whether the patient’s estimated cancer risk is >3% or not).

In a recent study that followed a signal detection approach, 216 GPs responded to 44 clinical vignettes online.^7^ The vignettes described patients for whom lung cancer could be considered. We constructed the vignettes using evidence from a retrospective case control study^8^ and estimated their 1-y cancer risk using a population risk prediction model (for details, see page 24 of Kostopoulou and colleagues^7^). In each vignette, respondents indicated whether they would refer the patient urgently. For each respondent, we calculated the rates of true-positive and false-positive responses (hit and false alarm rates) and used these to calculate the indices of SDT:

1. discrimination, that is, the physicians’ ability to discriminate patients who need to be referred urgently from those who do not (index *d′*), and
2. response bias, that is, the physicians’ propensity to refer patients urgently (index *c*).

We found average discrimination to be modest and highly variable. Discrimination did not relate to organizational performance (practice PPV and sensitivity). Response bias, on the other hand, was strongly related to organizational performance: physicians from practices with high PPV were less inclined to refer patients urgently than physicians from practices with lower PPV. We concluded that practices could achieve a high PPV if their physicians were reluctant to refer, thus reducing the chance of false-positive referrals, hence the denominator in the equation:

$$\mathrm{PPV}=\frac{\mathrm{True}\;\mathrm{positives}}{\mathrm{True}\;\mathrm{positives}+\mathrm{False}\;\mathrm{positives}}$$

In the absence of good discrimination, reluctance to refer cannot constitute good clinical practice.

We also found a gender effect, which we had hypothesized based on previous published research: an SDT study of transfer decisions for trauma patients found that male emergency physicians were less inclined than female emergency physicians to transfer patients to a trauma center.^9^ In our lung cancer SDT study, we found that male GPs were less inclined than female GPs to refer patients urgently to a specialist. However, our data were only suggestive of such an association (*P* = 0.04).^7(p28)^ Finally, we detected an inverse relationship between discrimination (*d′*) and physician experience (years in general practice), but this relationship was not linear. The experience variable was positively skewed, and we categorized it in 4 equal groups to perform the analyses. We found that the 2 groups with the least experience (0–6 y and 7–10 y, but not the group of 11–17 y) had significantly higher discrimination than the most experienced group (18–36 y). Moreover, inclination to refer dropped with increasing experience (see table 1 in ref. 7); however, we did not detect significant differences between experience groups.

**Table 1:** Frequencies of Urgent-Referral and No-Urgent-Referral Decisions across the 12,096 Responses

	Positive Cases	Negative Cases	Total
Urgent referrals	4474	2802	7276
No urgent referrals	1574	3246	4820
Total	6048	6048	12,096

## The Current Study: Aims

This study was carried out 1 y after the lung cancer study was completed. It had the following aims:

1. To test the generalizability of the relationship between organizational performance and physician discrimination and response bias by studying referral decisions for a different cancer (colorectal).
2. To test the stability of SDT indices (discrimination and response bias) for a given physician across cancers. In other words, is a physician equally discriminating in his or her referral decision making when seeing patients with possible cancer, irrespective of type of cancer? Is he or she equally inclined or disinclined to refer for suspected cancer, irrespective of cancer type?
3. To ascertain whether there are gender differences in response bias and to investigate a possible mediator. We hypothesized that if female GPs are indeed more inclined than male GPs to refer patients urgently for suspected cancer, this might be explained by women experiencing higher anxiety due to uncertainty. To measure this, we used the Stress from Uncertainty scale, one of the Physicians’ Reactions to Uncertainty (PRU) scales, which found gender differences in physicians.^10^ Stress from Uncertainty includes 2 subscales: Anxiety due to Uncertainty (5 items) and Concern about Bad Outcomes (3 items; Supplemental Appendix 1).
4. Our final aim was to improve the distribution of the experience variable for the analyses by recruiting more highly experienced GPs so that the relationship with SDT indices could be measured more reliably.

In our previous publication,^7(p22–23)^ we describe the basic tenets of SDT and how it applies to referral decision making of GPs. Therefore, we refrain from repeating these here.

## Method

### Sample Size and Recruitment

We wished to recruit the same number of GPs as in the lung cancer SDT study, that is, a minimum of 196 (the sample size calculation for the lung study is presented in Kostopoulou and colleagues,^7^ p 23–24). Furthermore, we wished to recruit the same GPs, as far as possible, to measure the stability (correlations) of *d′* and *c* across cancers. Thus, we first invited the GPs who had already participated in the lung study. We then invited the GPs who had volunteered to participate in the lung study but were unable to, as recruitment had closed. Finally, the National Institute for Health Research Clinical Research Network circulated our recruitment e-mail to general practices across England. In total, 428 GPs were invited (i.e., were sent the direct link to the study website): 214 of these had participated in the lung study, 147 had been on the lung study waiting list, and 67 highly experienced physicians (i.e., with ≥18 y in family practice) were invited following contact by the clinical research network.

We recruited an equal number of GPs from practices with high and low PPVs. We did not endeavor to do the same for practice sensitivity, as the lung cancer study showed no relationship with SDT indices. To dichotomize the PPV variable into high and low, we used the PPV data publicly available from Public Health England and followed the method described in previous publications.^5,7^

### The Vignettes

The vignettes described patients for whom colorectal cancer could be considered. To construct the vignettes, we used the online cancer risk calculator, QCancer (https://qcancer.org). QCancer uses the patient’s risk factors and symptoms to calculate the current probability of an undiagnosed cancer. The QCancer algorithm is based on electronic health record data of primary care patients visiting their GP.^11,12^ We constructed 48 clinical vignettes: brief descriptions of hypothetical patients with different combinations of demographics (age and sex), risk factors (body mass index, smoking, alcohol, and family history of gastrointestinal cancer), and symptoms associated with colorectal cancer that appear in the NICE guidelines (abdominal pain, weight loss, bowel symptoms [constipation or diarrhea], anemia, and rectal bleeding). Half of the vignettes required urgent referral (cancer risk >3%, “positive vignettes”) and half did not (cancer risk <3%, “negative vignettes”). Across the vignettes, the risk of colorectal cancer ranged from 0.18% to 18.04%, with a median 4.65% for positive vignettes and 1.30% for negative vignettes. The vignettes and their cancer risk are presented in Supplemental Appendix 2.

We used the Qualtrics platform (qualtrics.com) to present the vignettes online. To prevent response fatigue, we divided the vignettes in 2 equally sized sets (A and B), to be completed on different days, with at least 1 day in-between (see the Procedure section). Each set contained an equal number of positive and negative vignettes presented in a random order. The order in which the 2 sets were seen was counterbalanced across respondents, so that each set was seen first and second an equal number of times. To ensure that respondents maintained concentration, we included 4 “refocusing” clinical vignettes (2 per set), which appeared after 33% and 66% of the vignettes had been presented within each set. Refocusing vignettes had a different format, were unrelated to cancer or referral, and required a different type of response. They are not presented here.

### Procedure

Prospective participants were e-mailed a link to the study website. On the first screen, they read information about the study titled “Understanding Variation in Referral Decisions in Primary Care.” Cancer was not mentioned. Participants were asked to check a tick-box to provide informed consent before proceeding. They then saw 1 set of vignettes (either A or B). At each vignette, they were asked to choose 1 of 3 options (“refer to specialist urgently,”“refer to specialist routinely,”“not refer at this stage”) and give their working diagnosis (free-text box). Note that a routine referral does not involve a specific time frame; it can take several months before patients are seen by a specialist.

Twenty-four hours after the first set of vignettes was completed, a link to the second set was automatically e-mailed to participants. Twenty-four hours after the second set was completed, a link to the Stress from Uncertainty questionnaire was automatically e-mailed to participants. After completing it, GPs were reimbursed £120 for their participation and were sent a certificate of completion to include in their portfolio of continuous professional development. After all data were collected and analyzed, participants received individualized feedback, which contained the vignettes, the appropriate decisions, and their own decisions. It also contained their discrimination (index *d′*) and response bias (index *c*) with a brief explanation of each, as well as the mean *d′* and *c* of the whole sample, so that they could compare themselves to the “average” participant. GPs who had participated in both cancer SDT studies received feedback for both studies only after the colorectal cancer study was completed.

### Calculation of SDT Indices and Statistical Analyses

For each respondent, we calculated their hit rate (*H*), that is, the number of urgent referral decisions out of the 24 positive vignettes, and their false alarm rate (*FA*), that is, the number of urgent referral decisions out of the 24 negative vignettes. We derived *d′* and *c* directly from the respondents’ hit and false alarm rates, using the formulae

d′=z(H)−z(FA)

and

$$c=-\frac{1}{2}\left[z\left(H\right)+z\left(\mathrm{FA}\right)\right]$$

where *z* is the inverse of the cumulative normal distribution function.^13^ As some respondents had either no hits or no false alarms, precluding calculation of *d*′ and *c*, we added 0.5 to all data cells for all participants and used the corrected values for the calculations.^6,13^ After we had estimated each respondent’s *d′* and *c*, we regressed these on the 4 variables of interest (practice PPV, practice sensitivity, physician gender, and physician experience) in multivariable regressions.

We also estimated the SDT indices using a generalized linear modeling (GLM) approach.^14^ This does not require correction of 0 hits or false alarms and allows us to estimate the simultaneous effect of the 4 variables of interest on *d′* and *c* in a single regression model (see also appendix 2 in Kostopoulou et al.,^7^https://osf.io/qancs/). It also allows modeling of the nested structure of the data (multiple responses from each GP). The estimates and findings derived from the 2 distinct approaches should be comparable, although not necessarily identical.

## Results

Of the 428 GPs who were sent the study link, 254 (59%) completed the study but 2 of them only partially and were thus excluded from the analyses. The final sample comprised 252 physicians: 126 from high-PPV practices and 126 from low-PPV practices. Although we did not attempt to recruit equal numbers from high- and low-sensitivity practices, the final sample was relatively balanced in terms of sensitivity: 141 participants came from high-sensitivity practices and 111 from low-sensitivity practices.

Half of the participants were female (52%, 131/252). Of the total sample, 165 (65%) had participated in the lung study, 52 (21%) had been on the waiting list for that study, and 35 (14%) were recruited via the clinical research network. Participants came from 193 practices across England. The number of participants working in the same practice ranged from 1 to 4 (median 1). Experience ranged from 1 to 42 y in general practice (median 13 y, interquartile range >8 and <24 y) and was positively skewed (skewness 0.40, *P* = 0.009). Thus, we did not achieve our aim of a more normally distributed variable of physician experience.

Of the 12,096 decisions obtained (252 physicians responding to 48 vignettes), 7276 were urgent referrals (60.15%), 3796 were no referrals (31.38%), and 1024 were routine referrals (8.47%). For the analyses, we dichotomized the decision variable by collapsing the routine referrals and no referrals into 1 category (no urgent referral). Physicians correctly referred 74% of the positive vignettes urgently (i.e., missed 26%) and correctly rejected 54% of the negative vignettes (i.e., inappropriately referred 46%; Table 1). This is in contrast to the lung cancer SDT study, in which the rate of correct referrals was 46%, lower than the rate of correct rejections (77%).

### Discrimination (*d′*) and Response Bias (*c*) of the Sample and Comparisons between Cancers

The average *d′* was 0.79 (SD 0.32), ranging from 0.10 to 1.92 (median 0.77), and was comparable with that obtained in the lung cancer study (mean *d′* 0.77, SD 0.36, range −0.28 to 1.91, median 0.78).^7^

**Figure 1:**
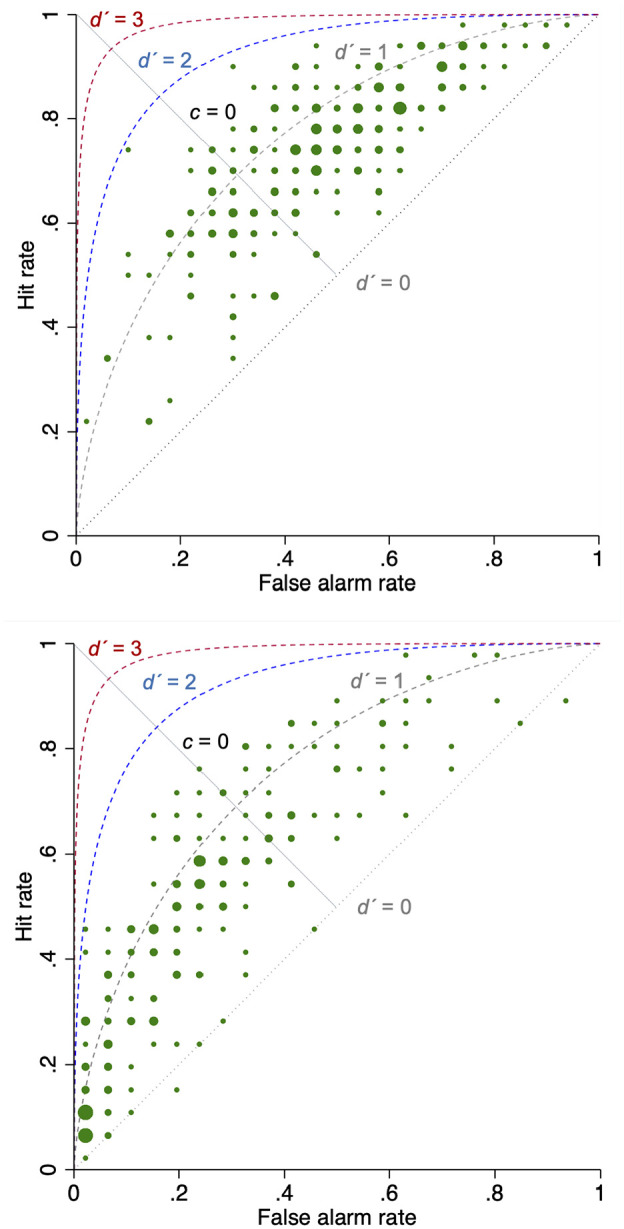
Top panel: Scatterplot of referral decision making in the colorectal cancer study (*N* = 252 GPs). Bottom panel: Scatterplot of referral decision making in the lung cancer study (*N* = 216 GPs).^7^ ^a^The scatterplots include theoretical Receiver Operating Characteristic curves at *d′* = 0, 1, 2, and 3. The dots are sized by frequency (number of physicians with the same hit and false alarm rates).

The average *c* was −0.29 (SD 0.50), ranging from −1.80 to 1.41 (median −0.31). Negative values of *c* suggest a lenient approach to referral; that is, the decision maker does not need much evidence to refer. In contrast, in the lung cancer study, physicians demonstrated a conservative approach to referral on average (mean *c* 0.50, SD 0.75, range −1.44 to 2.02, median 0.43).^7^ The difference in response bias between the 2 cancers is illustrated in Figure 1, where the Receiver Operating Characteristic curves from each study can be contrasted. As can be seen, discrimination (the cloud of dots representing physicians) is similar in both studies, whereas the response bias differs: in the colorectal cancer study (top panel), most respondents concentrate on the right of the diagonal that marks no response bias (*c* = 0, i.e., misses and false alarms are weighed equally), whereas in the lung cancer study (bottom panel), most respondents concentrate on the left of that diagonal. Indeed, in the colorectal cancer study, both the hit and false alarm rates were higher than in the lung cancer study (*H* = 0.74 and *FA* = 0.46 v. *H* = 0.45 and *FA* = 0.23 for colorectal and lung cancer studies, respectively).

The SDT estimates derived from the GLM approach were comparable with those derived using the traditional approach above and are presented in Supplemental Appendix 3.

### Stability of SDT Indices: Do Individual GPs Exhibit Similar Discrimination and Response Bias When Making Referral Decisions for Different Cancers?

To determine the stability of SDT indices, we performed Pearson correlations for *d′* and *c* for the GPs who had participated in both lung and colorectal SDT studies (*n* = 165). Discrimination (*d′*) did not correlate between the 2 studies (*r* = 0.01, *P* = 0.88), which might suggest that knowledge of the predictive value of symptoms is cancer specific. Response bias, on the other hand, was significantly correlated between the 2 studies (*r* = 0.39, *P* < 0.001). This means that if a GP was inclined to refer for one cancer, he or she was inclined to refer also for the other cancer, even though physicians as a group were more inclined to refer for colorectal than lung cancer. The significant correlation in *c* suggests that response bias is a relatively stable feature of physician referral decision making and does not depend on cancer type.

### Is There a Relationship between Organizational Performance and Physician Decision Making? Are There Gender-Related or Experience-Related Differences in Referral Decisions?

To measure relationships between organizational performance and physician decision making, and to test for gender-related and experience-related differences, we performed multivariable regressions of *d′* and *c* on practice PPV, practice sensitivity, GP gender, and GP experience.

Regressing *d′* on practice PPV, practice sensitivity, GP gender, and GP experience revealed no associations. Regressing *c* on these 4 predictors detected significant associations with practice PPV and physician gender only, echoing the results of the lung cancer study. Specifically, practice PPV was positively associated with *c* (b = 0.05 [0.03 to 0.07]; *P* < 0.001): as practice PPV increased, GPs’*c* also increased, that is, they were less inclined to refer. Female GPs had significantly lower *c* (were more inclined to refer) than their male counterparts (mean *c*−0.40 v. −0.17 for women vs. men, respectively, b = −0.17 [−0.30 to −0.05]; *P* = 0.006).

The GLM analytic approach produced similar results: practice PPV and GP gender were the only 2 significant factors in the mixed-effects probit regression model predicting urgent referral decisions. Furthermore, their associations with response bias were consistent with those described above (Supplemental Appendix 3).

### Is Stress from Uncertainty Responsible for Gender Differences in Response Bias?

Three women did not complete the Stress from Uncertainty scale. Women had significantly higher scores on the scale than men (means 30.41 v. 26.65, *t*_247_ = 3.51, *P* < 0.001). A linear regression detected a significant negative relationship between criterion (*c*) and stress from uncertainty, such that increasing Stress from Uncertainty scores were associated with lower *c*, that is, higher inclination to refer (b = −0.009 [−0.016 to −0.001]; *P* = 0.02). To determine whether stress from uncertainty explained the relationship between physician gender and response bias, we constructed a mediation model, with gender as the predictor, Stress from Uncertainty score as the mediator, and criterion (*c*) as the dependent variable. We found little evidence of mediation. Specifically, the direct effect of gender on *c* was significant (b = −0.21 [−0.33 to −0.08]; *P* = 0.001), as was the effect of gender on stress from uncertainty (b = 3.76 [1.67–5.85]; *P* < 0.001); the indirect effect of gender on *c* via stress from uncertainty was not significant (b = −0.023 [−0.052 to 0.007]; *P* = 0.135). The total effect of gender on *c* was −0.23 [−0.35 to −0.11], *P* < 0.001. Thus, the proportion of the total effect mediated was −0.023/−0.23 = 0.1 (10%). When we used the number of urgent referrals as the dependent variable, instead of *c*, the results were similar. Therefore, stress from uncertainty did not explain well why female physicians were more inclined to make urgent referrals than male physicians.

It is nevertheless likely that our sample size was too small to measure mediation. To determine the achieved power, we performed a post hoc Monte Carlo power analysis for indirect effects (https://schoemanna.shinyapps.io/mc_power_med/).^15^ We set the number of replications to 5000 and the Monte Carlo draws per replication to 20,000, and we used the default random seed of 1234 and the default 95% Monte Carlo confidence interval [CIs]. The values entered for the covariance matrix came from our data (pairwise correlations between the 3 variables and the variables’ standard deviations). A sample of 249 respondents achieves a power of only 34% (1 −*β*, which is the probability of correctly rejecting the null hypothesis). Thus, the probability of incorrectly retaining the null hypothesis is 66%. To achieve a power of 80%, the program estimated that 760 GPs would be needed. This is beyond the capacity of a single study and suggests that mediation, in this case, is too small to be of significance.

## Discussion

We conducted a signal detection study of physician referral decision making in cases of possible colorectal cancer, which enabled us to compare findings with a previous signal detection study in cases of possible lung cancer.^7^ It also allowed us to draw generalizable conclusions about physicians’ decision-making characteristics and their relationship with measures of organizational performance. The relationship between organizational performance (practice PPV) and physicians’ response bias (propensity to refer) was consistent and of similar magnitude in both studies. The lack of a relationship between organizational performance and GP discrimination was also a consistent finding. This consistency of findings increases our confidence in the validity of our results. Without good discrimination, a conservative referral approach can lead to missing cancers and diagnostic delays. A recent, national cohort study of 1.4 million cancer patients in England found evidence of an association between increased patient mortality with higher practice PPV.^3^ Although one cannot disentangle the causes of this association in this large cohort study, the relationship between practice PPV and GP response bias that we have consistently found is one possible explanation.

We also argue against the assumption that the publicly available measures of organizational performance accurately reflect the quality of clinical practice and, by extension, the quality of GP decision making. Organizational performance may be influenced by external factors entirely unrelated to GP decision making. For example, patients presenting to the emergency department without first visiting their GP would reduce practice sensitivity (i.e., the chance of cancer patients being diagnosed following urgent referral from their practice),^16^ which could perhaps explain the lack of a relationship between practice sensitivity and GP decision making in our 2 SDT studies.

The colorectal cancer study findings provide more convincing evidence of a relationship between GP gender and response bias, which was only suggestive in the lung cancer study. A recently published study that investigated associations between practice characteristics and rates of urgent cancer referrals reported a negative association between urgent referral rates and percentage of male GPs.^17^ This association was weak but statistically significant,^17(p577)^ and it would appear to add more credence to our findings. On the other hand, taken together, our 2 SDT studies do not provide any convincing or consistent evidence of a relationship between GP experience and either discrimination or response bias in cancer referral decision making.

Average discrimination was modest in both cancers. Arkes and Mellers^18^ reviewed a range of judgment tasks and their associated *d′.* For example, radiologists interpreting computed tomography (CT) scans had a *d′* of up to 2.9, while experts evaluating slides for cervical cancer achieved a *d′*of 1.6. The low average discrimination in our studies reflects the inherent uncertainty in cancer referral decision making, essentially, the low-positive predictive values of symptoms as indicators of cancer. Even a red flag symptom such as rectal bleeding has a sensitivity of 33% and a PPV of 2.9% for colorectal cancer.^19^

Discrimination was disease dependent; that is, there was no relationship between our physicians’ discrimination when dealing with lung cancer and their discrimination when dealing with colorectal cancer. This is likely to stem from the individuals’ idiosyncratic knowledge of how patient symptoms predict a cancer diagnosis. On the other hand, the correlation between a physician’s response bias when dealing with a series of possible lung cancer cases and his or her response bias when dealing with a series of colorectal cancer cases suggests that response bias is relatively stable and may influence other physician decisions that involve similar tradeoffs.

In comparison with the lung cancer study, this study found GPs to be much more inclined to refer cases of suspected colorectal cancer. Response bias depends on our perceived prevalence of an event and our perceived costs and benefits of the expected outcomes when responding “yes” versus “no.”^20^ For example, it is possible that physicians perceive colorectal cancer to be more common than lung cancer. They may also perceive that the benefits of a correct referral are higher (e.g., colorectal cancer patients are more likely to survive with treatment than lung cancer patients). In other words, they may perceive that the costs of a missed colorectal cancer are higher because colorectal cancer is more treatable than lung cancer. Lung cancer has been associated with both stigma (a smokers’ disease, thus self-inflicted) and the concept of “therapeutic nihilism,” whereby medical treatment is perceived to be of no value.^21,22^ There is also some evidence that primary care physicians are less likely to refer patients with advanced lung cancer than patients with advanced breast cancer.^23^ What about the perceived costs of an unnecessary referral? Since colonoscopy is more invasive than a lung CT scan, we would expect GPs to be more inclined to refer for the latter; however, this does not seem to be the case. It is possible that in these circumstances, physicians may discount the patient’s temporary discomfort from a diagnostic investigation. Clearly, these are post hoc hypotheses that our data cannot answer; a different study design would be required to investigate physicians’ motivations and concerns when referring patients to specialists for different diseases.

Lyratzopoulos and colleagues^24^ examined the number of consultations that preceded urgent referral for different cancers and equated diagnostic difficulty with ≥3 prereferral consultations. They then categorized cancers into 3 groups depending on the proportion of patients who were referred after ≥3 consultations: hard-to-suspect cancers (>30% of patents), cancers of intermediate difficulty (10%–30% of patients), and easier-to-suspect cancers (≤10 of patients). Lung cancer was deemed hard to suspect, whereas colorectal cancer was deemed of intermediate difficulty. We would argue, on the basis of our results, that the difference may be less related to diagnostic difficulty (which would manifest itself in different *d’s*) and more related to inclination to refer.

Scores on the subscale Anxiety due to Uncertainty of the PRU scale^10^ have been found to be positively associated with resource use in a Medicare health maintenance organization.^25^ Other studies, however, have not found an association between the PRU and health care costs.^26^ We found that stress from uncertainty (which included 2 subscales of the PRU: Anxiety due to Uncertainty and Concern about Bad Outcomes) was associated with more referrals and a greater propensity to refer; however, it did not explain satisfactorily why female physicians were more inclined to refer than male physicians, probably because of lack of statistical power.

Both SDT studies used frugal patient descriptions, devoid of the rich clinical and contextual information that a GP might elicit in real life. This was necessary for 2 reasons: 1) the signal detection approach requires respondents to make decisions on large numbers of cases, thus precluding long patient descriptions, and 2) the published evidence allowed only for specific symptoms and signs to be included in the vignettes, in order for risk to be estimated with precision (anything additional could have swayed decisions in unpredictable ways and harmed rather than helped GPs’ discrimination). It is also possible that additional considerations, such as the availability of diagnostic services locally, may influence referral decisions in real life.^4^ Our aim was to measure response tendencies rather than predict how a specific GP would respond to a specific vignette patient in real life, having access to richer information or being subject to local constraints.

Despite their limitations, decision-making studies based on a signal detection approach are invaluable for measuring separately the 2 aspects of decision making that other approaches conflate, namely, discrimination and response bias. This is particularly important, as these 2 aspects respond to different improvement measures (e.g., training v. incentives). Signal detection studies are also useful for generating data at the level of the individual; when such data are not available, organizations have to rely on aggregate-level data to monitor and regulate performance, which could lead to the wrong conclusions. However, SDT studies need to be supplemented by other study designs to answer questions about the habits, motivations, concerns, and expectations of decision makers, which underlie their response bias, as well as the contribution of knowledge versus state of the evidence that determine discrimination.

## Supplemental Material

Appendix_1_online_supp – Supplemental material for Disentangling the Relationship between Physician and Organizational Performance: A Signal Detection Approach Supplemental material, Appendix_1_online_supp for Disentangling the Relationship between Physician and Organizational Performance: A Signal Detection Approach by Olga Kostopoulou, Martine Nurek and Brendan C. Delaney in Medical Decision Making

Appendix_2_online_supp – Supplemental material for Disentangling the Relationship between Physician and Organizational Performance: A Signal Detection Approach Supplemental material, Appendix_2_online_supp for Disentangling the Relationship between Physician and Organizational Performance: A Signal Detection Approach by Olga Kostopoulou, Martine Nurek and Brendan C. Delaney in Medical Decision Making

Appendix_3_online_supp – Supplemental material for Disentangling the Relationship between Physician and Organizational Performance: A Signal Detection Approach Supplemental material, Appendix_3_online_supp for Disentangling the Relationship between Physician and Organizational Performance: A Signal Detection Approach by Olga Kostopoulou, Martine Nurek and Brendan C. Delaney in Medical Decision Making

## References

[1] ecancer News. World Cancer Day 2019: global cancer experts call for urgent action to improve early cancer detection. Available from: https://ecancer.org/news/15350-world-cancer-day-2019—global-cancer-experts-call-for-urgent-action-to-improve-early-cancer-detection.php. Published 2019. Accessed May 29, 2019.

[2] Allemani C Matsuda T Carlo V Di , et al. Global surveillance of trends in cancer survival 2000–14 (CONCORD-3): analysis of individual records for 37 513 025 patients diagnosed with one of 18 cancers from 322 population-based registries in 71 countries. Lancet. 2018;391(10125):1023–75. 10.1016/S0140-6736(17)33326-3 PMC5879496 29395269

[3] Round T Gildea C Ashworth M. Association between use of urgent suspected cancer referral and mortality and stage at diagnosis: a 5-year national cohort study. Br J Gen Pract. 2020;70(695):e389–98. 10.3399/bjgp20X709433 PMC7176359 32312762

[4] Burton C O’Neill L Oliver P Murchie P. Contribution of primary care organisation and specialist care provider to variation in GP referrals for suspected cancer: ecological analysis of national data. BMJ Qual Saf. 2019;29(4):274–6. 10.1136/bmjqs-2019-009469 31586938

[5] Meechan D Gildea C Hollingworth L Richards MA Riley D Rubin G. Variation in use of the 2-week referral pathway for suspected cancer: a cross-sectional analysis. Br J Gen Pract. 2012;62(602):e590–7. 10.3399/bjgp12X654551 PMC3426597 22947579

[6] Macmillan N Creelman C. Detection Theory: A User’s Guide. 2nd ed. New York: Lawrence Erlbaum Associates; 2005.

[7] Kostopoulou O Nurek M Cantarella S Okoli G Fiorentino F Delaney BC. Referral decision making of general practitioners: a signal detection study. Med Decis Mak. 2019;39(1):21–31. 10.1177/0272989X18813357 PMC6311616 30799690

[8] Hamilton W. The CAPER studies: five case-control studies aimed at identifying and quantifying the risk of cancer in symptomatic primary care patients. Br J Cancer. 2009;101(suppl):S80–6. 10.1038/sj.bjc.6605396 PMC2790706 19956169

[9] Mohan D Rosengart MR Farris C Fischhoff B Angus DC Barnato AE. Sources of non-compliance with clinical practice guidelines in trauma triage: a decision science study. Implement Sci. 2012;7:103. 10.1186/1748-5908-7-103 PMC3503726 23098291

[10] Gerrity MS White KP DeVellis RF Dittus RS. Physicians’ reactions to uncertainty: refining the constructs and scales. Motiv Emot. 1995;19(3):175–91.

[11] Hippisley-Cox J Coupland C. Symptoms and risk factors to identify men with suspected cancer in primary care: derivation and validation of an algorithm. Br J Gen Pract. 2013;63(606):e1–10. 10.3399/bjgp13X660724 PMC3529287 23336443

[12] Hippisley-Cox J Coupland C. Symptoms and risk factors to identify women with suspected cancer in primary care: derivation and validation of an algorithm. Br J Gen Pract. 2013;63(606):e11–21. 10.3399/bjgp13X660733 PMC3529288 23336450

[13] Stanislaw H Todorov N. Calculation of signal detection theory measures. Behav Res Methods Instruments Comput. 1999;31(1):137–49. 10.3758/bf03207704 10495845

[14] DeCarlo LT. Signal detection theory and generalized linear models. Psychol Methods. 1998;3(2):186–205.

[15] Schoemann AM Boulton AJ Short SD. Determining power and sample size for simple and complex mediation models. Soc Sci Personal Sci. 2017.

[16] Abel GA Shelton J Johnson S Elliss-Brookes L Lyratzopoulos G. Cancer-specific variation in emergency presentation by sex, age and deprivation across 27 common and rarer cancers. Br J Cancer. 2015;112:S129–36. 10.1038/bjc.2015.52 PMC4385986 25734396

[17] Mendonca SC Abel GA Gildea C , et al. Associations between general practice characteristics with use of urgent referrals for suspected cancer and endoscopies: a cross-sectional ecological study. Fam Pract. 2019;36(5):573–80. 10.1093/fampra/cmy118 PMC6781939 30541076

[18] Arkes HR Mellers BA. Do juries meet our expectations? Law Hum Behav. 2002;625:632–33. 10.1023/a:1020929517312 12508698

[19] Hippisley-Cox J Coupland C. Identifying patients with suspected colorectal cancer in primary care: derivation and validation of an algorithm. Br J Gen Pract. 2012;62(594):e29–37. 10.3399/bjgp12X616346 PMC3252537 22520670

[20] Swets JA Dawes RM Monahan J. Psychological science can improve diagnostic decisions. Psychol Sci Public Interes. 2000;1(1):1–26. 10.1111/1529-1006.001 26151979

[21] Sriram N Mills J Lang E , et al. Attitudes and stereotypes in lung cancer versus breast cancer. PLoS One. 2015;10(12):1–13. 10.1371/journal.pone.0145715 PMC4689531 26698307

[22] Hamann HA Lee JW Schiller JH , et al. Clinician perceptions of care difficulty, quality of life, and symptom reports for lung cancer patients: an analysis from the Symptom Outcomes and Practice Patterns (SOAPP) study. J Thorac Oncol. 2013;8(12):1474–83. 10.1097/01.JTO.0000437501.83763.5d PMC3936653 24189514

[23] Wassenaar TR Eickhoff JC Jarzemsky DR Smith SS Larson ML Schiller JH. Differences in primary care clinicians’ approach to non-small cell lung cancer patients compared with breast cancer. J Thorac Oncol. 2007;2(8):722–8. 10.1097/JTO.0b013e3180cc2599 17762338

[24] Lyratzopoulos G Wardle J Rubin G. Rethinking diagnostic delay in cancer: how difficult is the diagnosis? BMJ. 2014;349:g7400. 10.1136/bmj.g7400 25491791

[25] Allison JJ Kiefe CI Cook EF Gerrity MS Orav EJ Centor R. The association of physician attitudes about uncertainty and risk taking with resource use in a Medicare HMO. Med Decis Mak. 1998;18(3):320–9. 10.1177/0272989X9801800310 9679997

[26] Fiscella K Franks P. Is patient HMO insurance or physician HMO participation related to racial disparities in primary care? Am J Manag Care. 2005;11(6):397–402. 15974559

