# Supplementary material for: Disentangling the Relationship between Physician and Organizational Performance: A Signal Detection Approach
Source: Med Decis Making. 2020 Jul 1;40(6):746–55. doi: 10.1177/0272989X20936212 (PMC7457451; doi:10.1177/0272989X20936212)
Supplement: Appendix_1_online_supp – Supplemental material for Disentangling the Relationship between Physician and Organizational Performance: A Signal Detection Approach [file Appendix_1_online_supp.docx]

**Appendix 1: “Stress from Uncertainty” scale from the “Physicians’ Reactions to Uncertainty” (PRU) scales (Gerrity, White, DeVellis & Dittus, 1995).**

1. I usually feel anxious when I am not sure of a diagnosis.
2. I find the uncertainty involved in patient care disconcerting.
3. Uncertainty in patient care makes me uneasy.
4. I am quite comfortable with the uncertainty in patient care. (RS)
5. The uncertainty of patient care often troubles me.
6. When I am uncertain of a diagnosis, I imagine all sorts of bad scenarios -- patient dies, patient sues, etc.
7. I fear being held accountable for the limits of my knowledge.
8. I worry about malpractice when I do not know a patient's diagnosis.

Note: Items 1-5 measure the construct “Anxiety due to Uncertainty” (Cronbach’s alpha 0.86); items 6-8 measure the construct “Concern About Bad Outcomes” (Cronbach’s alpha 0.73).
