# Supplementary material for: Disentangling the Relationship between Physician and Organizational Performance: A Signal Detection Approach
Source: Med Decis Making. 2020 Jul 1;40(6):746–55. doi: 10.1177/0272989X20936212 (PMC7457451; doi:10.1177/0272989X20936212)
Supplement: Appendix_2_online_supp – Supplemental material for Disentangling the Relationship between Physician and Organizational Performance: A Signal Detection Approach [file Appendix_2_online_supp.docx]

**Appendix 2: The 48 vignettes**

**Positive vignettes**: cancer risk >3%

1. Holly Hunt is 76 years old. She has a BMI of 24.3. She has never smoked and drinks 7 units of alcohol per week. Today, she comes in to get the results of her recent blood test: it shows microcytic anaemia (Hb 10.5) with low ferritin. She also mentions that she has lost some weight recently. She does not weigh herself but noticed that her skirts are loose. (cancer risk 3.22%)
2. Bob Jacobson is 65 years old. He is a heavy smoker (25 cigarettes a day) and drinks 14 units of alcohol per week. His BMI is 24.2. He comes to get the results of his recent blood test: it shows microcytic anaemia (Hb 10.9) with low ferritin. He also mentions that his motions have become looser in the past month, and that he needs to go to the loo more frequently than his usual once a day. (cancer risk 4.05%)
3. Ewelina Jankowska is 70. She has never smoked but she drinks alcohol occasionally, about 7 units a week. Her BMI is 26.4. Ewelina comes to see you because she has been experiencing abdominal pain for one month. A recent, unrelated blood test shows microcytic anaemia (Hb 10.8) with low ferritin. (cancer risk 4.07%)
4. Lilly Wallace is 78 years old. She is an ex-smoker and drinks about 5 units of alcohol per week. Her BMI is 21.3. She comes to see you with constipation during the last month. She also mentions that she has lost weight, which her husband noticed and commented upon. You order a full blood count, which shows microcytic anaemia (Hb 10.1) with low ferritin. (cancer risk 4.60%)
5. Rose Edwards is 52. She has never smoked and drinks 14 units of alcohol per week. Her BMI is 24.3. Today, she consults because she’s had some rectal bleeding in the past month. This worries her, because her mother has been diagnosed with gastrointestinal cancer. Rose also tells you that she has lost weight, which her husband noticed and remarked upon. (cancer risk 4.85%)
6. Louise Slaven is 69 years old and overweight (BMI of 30). She smokes only socially (about 3 cigarettes a day) and drinks 7 units of alcohol per week. A few months ago, she had a blood test that showed microcytic anaemia (Hb 10.7) with low ferritin. She did not come back to see you about this. Today, she comes in complaining about constipation and abdominal pain that she’s had in the last month. (cancer risk 5.08%)
7. Antonio DiMarco is 78. He smokes about 12 cigarettes a day and drinks no alcohol. His BMI is 24.2. He comes to see you because he's lost some weight recently without dieting. His wife remarked upon it. Antonio also mentions that he’s been passing stool more frequently than usual, which worries him a little because his father died of gastrointestinal cancer. (cancer risk 5.16%)
8. Harold Schoof is 72. His BMI is 22.8. He has never smoked and rarely drinks alcohol (on average, 2 units per week). He comes to see you about abdominal pain that he has had for about 4 weeks. He also mentions that his stools are more loose than usual. (cancer risk 5.19%)
9. Henry Lipp is 75 years old. He has never smoked and drinks alcohol occasionally, about 4 units per week. His BMI is 24.9. He is consulting because he noticed some blood in his motions and got worried. He has no other symptoms. (cancer risk 6.33%)
10. Joshua Smith is 66 years old, with a BMI of 27.1. He smokes on average 9 cigarettes a day, and drinks 14 units of alcohol per week. Joshua has been having constipation for the last month. He’s also had some rectal bleeding, which made him decide to come and see you today. (cancer risk 6.76%)
11. William Tallent is 73 years old, and has never smoked in his life. His BMI is 23.1. He drinks 7 units of alcohol per week. Today he comes to see you complaining about abdominal pain that he has had for about a month. He also says that his stools are looser than usual and that he has lost weight. (cancer risk 15.93%)
12. Miriam Maisel is 79 years old, with a BMI of 20.7. In the past, she used to smoke. She never drinks alcohol. Miriam tells you that, in the past month, she has been having abdominal pain and has been passing stool more frequently than usual. She is also concerned about having lost weight; her dresses feel loose. You order a full blood count; it shows microcytic anaemia (Hb 7.3) with low ferritin. (cancer risk 18.04%)
13. Joseph Cohen is 77 years old, with a BMI of 22.8. He has never smoked in his life and drinks 7 units of alcohol weekly. He comes in to see you about an abdominal pain that he’s been having for about a month. This worries him because his father died of gastrointestinal cancer. You ask whether he has any other symptoms, and he tells you that he does not. (cancer risk 3.17%)
14. Walter Lourenco is 58 years old, with a BMI of 24.4. He smokes a packet of cigarettes a day, and drinks 22 units of alcohol per week. He comes to see you because he’s had abdominal pain for the past month. Upon questioning, Walter mentions that he’s been passing stool more frequently than usual. (cancer risk 3.27%)
15. Isabella Tarragona is 39 years old. Her BMI is 26.4. She smokes half a packet of cigarettes a day and drinks 21 units of alcohol per week. Today, she comes to see you complaining of abdominal pain and loose stool that she’s had for about a month. This worries her because her brother was recently diagnosed with gastrointestinal cancer. She also mentions that she’s lost some weight in the past few weeks, which her husband commented upon. You order a blood test that shows microcytic anaemia (Hb 10.2) with low ferritin. (cancer risk 3.32%)
16. Liz Robinson is 57 years old. She smokes 5 cigarettes a day, and drinks 21 units of alcohol per week. Her BMI is 25.5. She comes to see you because she has lost some weight recently, which her husband remarked upon. She also tells you that she has been passing stool more frequently than usual. You order a FBC, which shows microcytic anaemia (Hb 10.2) with low ferritin. (cancer risk 3.36%)
17. Paul Taylor is 39 years old, with a BMI of 26.1. He used to smoke, but not anymore. His alcohol intake is 21 units per week. Paul tells you that, in the past month, he has been having abdominal pain and has been passing stool more frequently than usual. He is also concerned about having lost weight; his trousers feel loose. You order a full blood count; it shows microcytic anaemia (Hb 10.9) with low ferritin. (cancer risk 3.37%)
18. Daniel Fox is 59, with a BMI of 27.1. He has never smoked in his life and drinks 22 units of alcohol per week. He lives alone – he used to live with his mother (who needed full-time care), but she recently died of gastrointestinal cancer. He comes to see you because he has lost some weight recently, which his sister noticed and told him to speak to the GP. Upon questioning, Daniel mentions that he’s been passing stool more frequently than usual. He has no other symptoms. (cancer risk 3.65%)
19. Raymond Stone is 59 years old and obese (BMI 33.3). He used to smoke, but not anymore. He drinks 25 units of alcohol per week. He comes to see you because he’s had abdominal pain for the past month. Upon questioning, Raymond mentions that his stools have been looser than usual. (cancer risk 3.91%)
20. Vivienne Chasen is 58 and has a BMI of 24.8. She used to smoke (not anymore) and drinks 21 units of alcohol per week. Today, she is complaining that she has had abdominal pain for almost a month. She is worried about this because her sister was diagnosed with gastrointestinal cancer. You notice in her record that a recent blood test showed microcytic anaemia (Hb 10.7) with low ferritin. (cancer risk 4.14%)
21. Debbie Lawrence is 58 years old. She smokes socially (about 3 cigarettes a day) and drinks alcohol occasionally, about 3 units a week. Her BMI is 21.8. She comes in complaining of abdominal pain that she has had for more than a month. She has also noticed that she is passing stool more frequently than usual (2 or 3 times a day). You order a blood test that shows microcytic anaemia (Hb 10.3) with low ferritin. (cancer risk 4.70%)
22. Lorna Dirst is 59, with a BMI of 20.9. She has never smoked and drinks 7 units of alcohol per week. She comes to see you complaining of abdominal pain and loose stool, which she first noticed about a month ago. You order a blood test that shows microcytic anaemia (Hb 10.4) with low ferritin. (cancer risk 5.04%)
23. Ben Goldin is 55 years old, with a BMI of 25.1. He smokes socially (about 3 cigarettes a day), and drinks 14 units of alcohol per week. He comes to see you because he has lost some weight recently, which his daughter commented upon. Upon questioning, he also mentions that his stools are looser than usual. You order a FBC, which shows microcytic anaemia (Hb 10.9) with low ferritin. (cancer risk 5.30%)
24. Jane Dobbs is 59 years old. She used to smoke (not anymore) and drinks 21 units of alcohol weekly. Her BMI is 25.5. She comes in complaining of abdominal pain that she has had for about 4 weeks. She has also noticed that her stools are looser than usual. You order a blood test that shows microcytic anaemia (Hb 10.4) with low ferritin. (cancer risk 6.01%)

**Negative vignettes**: cancer risk <3%

1. Laura Levin is 39 years old, and an ex-smoker. She drinks about 22 units of alcohol per week and her BMI is 30.4. She comes to get the results of a recent blood test: it shows microcytic anaemia (Hb 10.6) with low ferritin. She also mentions that her motions have become looser in the past month, and that she needs to go to the loo more frequently than her usual once a day. (cancer risk 0.18%)
2. Bryony Barnes is 56 and overweight (BMI of 30). She smokes 25 cigarettes a day, and drinks 21 units of alcohol per week. She comes to see you because she has had constipation in the past month. Bryony also tells you that she’s lost some weight recently without dieting. (cancer risk 0.49%)
3. Adam Harper is 57 years old. He has never smoked in his life, and drinks about 21 units of alcohol a week. His BMI is 24.6. Adam noticed that recently his motions have become looser and that he needs to go to the loo more frequently than his usual once per day. He says that he hasn’t changed his diet and that he has no other symptoms. (cancer risk 0.69%)
4. Matt Crayton is 75 years old, with a BMI of 24.4. He has never smoked cigarettes. He drinks alcohol only occasionally (about 4 units per week). Matt consults because he has lost some weight recently. His friends noticed and told him to see the GP. Matt says that he has not changed his diet and has no other symptoms. (cancer risk 1.08%)
5. Robert Barclay is 49 years old. He has never smoked in his life, and drinks about 14 units of alcohol per week. His BMI is 20.9. Recently, Robert noticed some blood in his motions and decided to seek your advice. He has no other symptoms. (cancer risk 1.18%)
6. Irene Marple is 59 years old. She used to smoke when she was younger but not anymore. Her alcohol intake is 14 units per week. Her BMI is 22.4. She comes to see you because she’s had abdominal pain for the past month. Upon questioning, Irene mentions that she’s been passing stool more frequently than usual. (cancer risk 1.25%)
7. Lucy Lannister is 57 years old, and has a BMI of 24.2. She has never smoked and rarely drinks alcohol (on average, 1 unit per week). Today, she comes in with her husband to get the results of her recent blood test: it shows microcytic anaemia (Hb 10.7) with low ferritin. Her husband then mentions that Lucy has lost some weight recently. (cancer risk 1.35%)
8. Nina Durbridge is 54 and has a BMI of 27.2. She used to smoke but not anymore. She drinks about 21 units of alcohol per week. Nina comes in for the results of her recent blood test: it shows microcytic anaemia (Hb 10.8) with a low ferritin. Upon questioning, she tells you that she has had abdominal pain for about a month. (cancer risk 2.09%)
9. Bert Harris is 77 years old. He has never smoked in his life, and drinks 8 units of alcohol per week. His BMI is 19.8. He comes to see you today because he has been having abdominal pain for about a month, but no other symptoms. (cancer risk 2.10%)
10. Olu Ojukwu is 76 years old, and has a BMI of 27.1. He has never been a smoker, and drinks about 7 units of alcohol a week. He has been experiencing abdominal pain for the last month, and comes in to ask your advice. He has no other symptoms. (cancer risk 2.16%)
11. Valerie Shields is 56 years old. She is a light smoker of about 5 cigarettes a day, and drinks 14 units of alcohol per week. Her BMI is 21.8. Her son noticed that she lost weight recently, and advised her to see the GP. Valerie says that she has also been passing stool more frequently than usual. You order a FBC, which shows microcytic anaemia (Hb 10.2) with low ferritin. (cancer risk 2.60%)
12. Jessica Fortes is 57. She smokes a packet of cigarettes a day and drinks 14 units of alcohol per week. Her BMI is 25.9. She comes to see you complaining of abdominal pain and constipation for 4 weeks. She also had an unrelated blood test last month. It showed microcytic anaemia (Hb 10.7) with low ferritin. (cancer risk 2.83%)
13. Darryl Burton is 44 years old, and has a BMI of 22.8. He has never smoked cigarettes and limits his alcohol intake to 7 units per week. He has made an appointment to see you because he’s been having abdominal pain in the past month. He also mentions that he’s lost some weight, his clothes feel loose. (cancer risk 0.46%)
14. Patricia Staunton is 47 years old. She used to smoke (not anymore) and drinks 14 units of alcohol per week. Her BMI is 26.4. Her sister told her to come and see you because she lost some weight recently. She is also complaining of abdominal pain in the last month. (cancer risk 0.58%)
15. Tony Dallas is 64 years old. He has never smoked in his life, and drinks about 3 units of alcohol per week. His BMI is 23.8. Tony tells you that his motions have become looser, and that he needs to go to the loo more frequently than his usual once per day. Tony has no other symptoms. (cancer risk 0.82%)
16. Polly Penkin is 52 years old, with a BMI of 22.4. She smokes 20 cigarettes a day and drinks no alcohol. Today, she comes in to ask your advice about some blood that she noticed in her motions in the past month. She has no other symptoms. (cancer risk 0.96%)
17. Conrad Kerr is 62 years old. He smokes 5 cigarettes a day and drinks about 22 units of alcohol per week. His BMI is 27.1. His wife told him to come and see you because he lost some weight recently. He is also complaining of constipation in the last month. (cancer risk 1.04%)
18. Eleanor Ward is 48 years old, with a BMI of 25.9. She smokes on average 3 cigarettes a day, and drinks 4 units of alcohol per week. Eleanor has been having constipation for the last month. She’s also had some rectal bleeding, which made her decide to come and see you today. (cancer risk 1.15%)
19. Donna Draper is 62 years old, with a BMI of 27.2. She has never smoked and her alcohol intake is 7 units per week. She comes to get the results of her recent blood test: it shows microcytic anaemia (Hb 10.4) with low ferritin. She also mentions that her motions have become looser in the past month, and that she needs to go to the loo more frequently than her usual once a day. (cancer risk 1.40%)
20. Mike Ainsley is 68. He has a BMI of 24.4. He smokes half a packet of cigarettes a day and drinks 14 units of alcohol per week. He comes to see you because he has been experiencing abdominal pain and constipation in the last four weeks. (cancer risk 1.88%)
21. Lynne Green is 61, with a BMI of 23.3. She used to smoke, but she has quit. Her alcohol intake is 7 units per week. She comes to see you, complaining of constipation that she’s had for a month. Upon questioning, she mentions that she’s lost some weight recently, which her family noticed too. You order a FBC, which shows microcytic anaemia (Hb 10.8) with low ferritin. (cancer risk 2.35%)
22. Jack Barton is 45. He is an ex-smoker and drinks 21 units of alcohol per week. His BMI is 24.6. Today, he consults because he’s had some rectal bleeding in the past month. He also tells you that he has lost weight, which his wife noticed and commented upon. (cancer risk 2.42%)
23. Albert Newton is 60 years old. He has a BMI of 22.3. He smokes occasionally (on average, 1 cigarette a day) and he does not drink alcohol. He comes to see you because he has been experiencing abdominal pain in the last four weeks. He has also noticed that his stools are looser than usual. (cancer risk 2.47%)
24. Rita Flott is 60 years old. Her BMI is 21.4. She has never smoked in her life and drinks no alcohol. A few months ago, she had a blood test that showed microcytic anaemia (Hb 10.7) with low ferritin. She did not come back to see you about this. Today, she comes in complaining about constipation and abdominal pain that she’s had in the last month. (cancer risk 2.87%)
