# Supplementary material for: Disentangling the Relationship between Physician and Organizational Performance: A Signal Detection Approach
Source: Med Decis Making. 2020 Jul 1;40(6):746–55. doi: 10.1177/0272989X20936212 (PMC7457451; doi:10.1177/0272989X20936212)
Supplement: Appendix_3_online_supp – Supplemental material for Disentangling the Relationship between Physician and Organizational Performance: A Signal Detection Approach [file Appendix_3_online_supp.docx]

**Appendix 3: A GLM-based estimation of SDT**

An alternative method of estimating SDT indices is to use a Generalized Linear Modeling approach. This enables us to enter covariates in the regression model and see their simultaneous effect on *d´* and *c*, rather than running separate regression models for each SDT index. Using probit regression, we can regress the decision (urgent referral vs. no urgent referral) on vignette type (positive vs. negative). The slope of the model quantifies the increase in the z-score (the standardized probability) of a referral decision when the case is positive – this is equal to *d´*. The intercept is a measure of response bias (*-k*): it estimates the standardized probability of a false alarm, *z(FA).* See also, Kostopoulou et al. 2019, Appendix 2 (https://osf.io/qancs/).

We conducted a mixed effects probit regression with random intercept for respondents and random slopes for vignette type; this allows respondents to differ both in their response bias and discrimination. The slope of the model was 0.81 (95% CI 0.76-0.86, *P*<0.0001). This is comparable to the average discrimination of the physician sample (*d´* = 0.79), estimated using the traditional method based on each GP’s hit and false alarm rates.

The intercept of the model (*-k*) was -0.10 (95% CI -0.17 to -0.03, p=0.003), thus *k* = 0.10. From this, we can calculate the criterion location (*c*), i.e., the distance from the neutral point (where there is no bias, i.e., misses and false alarms are weighted equally), using the formula: *k* = *c* + *d´*/2. Thus, *c* = *k* - *d´*/2 = 0.10 - (0.81/2) = 0.10 - 0.405 = -0.305. This model estimate is also very close to the average *c* estimated using the traditional method (*c* = -0.29).

|  | Coefficient | 95% CI | P |
| --- | --- | --- | --- |
| Vignette type  (negative=0, positive=1) | 0.81 | 0.76 to 0.86 | 0.000 |
| Constant | -0.10 | -0.17 to -0.03 | 0.003 |

Table 1. Probit regression coefficients (95% CI and P values) for predicting response

probability (urgent referral vs. no urgent referral) as a function of vignette type (positive vs. negative)

Regression model estimates for the intercept (-0.10) and the slope (0.81) give respectively *z(FA)* = -0.10 and *z(H)* = 0.71 (from *d*´= *z*(*H*)-*z*(*FA*)). Converting into probabilities, we get *p(FA)* = 0.46 and *p(H)* = 0.76, which match well the mean hit and false alarm rates of the data reported in the main body of the manuscript (0.46 and 0.74 respectively).

We entered simultaneously the four covariates of interest (practice PPV, practice sensitivity, physician gender, and physician experience) in the probit regression model. Only practice PPV and physician gender were significantly associated with the referral decision: increasing practice PPV was associated with decreasing chance of referral (b = -0.05 [-0.07 to -0.03] *P*<0.001); female physicians were more likely to refer than males (b = 0.18 [0.06 to 0.30] *P*=0.004).

|  | Coefficient | 95% CI | P |
| --- | --- | --- | --- |
| Vignette type  (negative=0, positive=1) | 0.81 | 0.76 to 0.86 | 0.000 |
| Practice PPV | -0.05 | -0.07 to -0.03 | 0.000 |
| Practice sensitivity | 0.004 | -0.004 to 0.13 | 0.30 |
| Physician gender  (male=0, female=1) | 0.18 | 0.06 to 0.30 | 0.004 |
| Physician experience | -0.0003 | -0.007 to 0.006 | 0.93 |
| Constant | -0.19 | -0.28 to -0.10 | 0.000 |

Table 2. Probit regression coefficients (95% CI and P values) for predicting response

probability (urgent referral vs. no urgent referral) as a function of vignette type (positive vs. negative), practice PPV, practice sensitivity, physician gender and physician experience.
